# Comparison of the effects of mixed tempeh with soy tempeh on cognitive function in older people

**DOI:** 10.3389/fnut.2025.1551211

**Published:** 2025-05-12

**Authors:** Yuda Turana, Yvonne Suzy Handajani, Tati Barus, Kevin Kristian, Elvina Theodoraliu, Ika Suswanti

**Affiliations:** ^1^Department of Neurology, School of Medicine and Health Sciences, Atma Jaya Catholic University of Indonesia, Jakarta, Indonesia; ^2^Center of Health Research, Atma Jaya Catholic University of Indonesia, Jakarta, Indonesia; ^3^School of Bioscience, Technology, and Innovation, Atma Jaya Catholic University of Indonesia, Jakarta, Indonesia; ^4^Center for Tempeh Research and Development School of Bioscience, Technology, and Innovation, Atma Jaya Catholic University of Indonesia, Jakarta, Indonesia; ^5^Department of Public Health and Nutrition, School of Medicine and Health Sciences, Atma Jaya Catholic University of Indonesia, Jakarta, Indonesia; ^6^School of Medicine and Health Sciences, Atma Jaya Catholic University of Indonesia, Jakarta, Indonesia

**Keywords:** adzuki beans, cognitive, pumpkin seeds, older people, sunflower seeds, tempeh

## Abstract

**Introduction:**

Antioxidants may help alleviate cognitive impairment in older adults, which is often caused by oxidative stress. This research focuses on developing tempeh enriched with antioxidant-rich ingredients, including sunflower seeds, pumpkin seeds, and adzuki beans, to enhance its neuroprotective properties. This study is the first to investigate the effectiveness of mixed tempeh in reducing cognitive decline.

**Methods:**

This experimental study (a non-randomized controlled trial) included 57 older participants with mild cognitive impairment who did not have diabetes. The participants were divided into two groups: one consumed mixed tempeh (comprising sunflower seeds, pumpkin seeds, adzuki beans, and regular soybeans), while the other group consumed soy tempeh. Both groups were instructed to consume 100 g daily over a period of 4 months and to avoid other fermented foods. Cognitive assessments were conducted before and after the intervention to evaluate the effects of tempeh consumption.

**Results:**

The majority of participants were female (68.4%), aged over 65 years (77.2%), and had an education level of 12 years or more (59.6%). The mixed tempeh group showed improvement in three cognitive domains (global cognitive, memory, and verbal fluency) before and after the intervention, while the soy tempeh group experienced improvements in two domains (memory and visuospatial).

**Conclusion:**

The study highlights the cognitive benefits of tempeh, particularly when mixed with other nutrient-rich ingredients. The combination of sunflower seeds, pumpkin seeds, and adzuki beans in mixed tempeh provides superior neuroprotective effects than traditional soy tempeh. This research supports the idea of promoting mixed tempeh as a healthy food alternative, especially for older adults, by offering enhanced nutritional value and cognitive health benefits.

## 1 Introduction

As the aging population expands, increasing focus has been placed on health issues related to aging, with cognitive impairment being a leading cause of diminished quality of life for older people. The prevalence of cognitive impairment varies between 5.1 and 41.0%, depending on the research methodology, diagnostic criteria, and cognitive assessment tools (1). This condition is characterized by memory loss, learning difficulties, and a decreased concentration ability (2). Consequently, it results in the individual’s loss of autonomy, dependence, and a heightened need for long-term caregivers and healthcare services (1).

Mechanisms such as oxidative stress and free radical damage mainly contribute to aging-related cognitive decline. Due to various genetic or environmental factors, these mechanisms get aggravated in pathological conditions (3). The oxidative stress theory of aging proposes that age-related cognitive deterioration is driven by oxidative damage to macromolecules, such as DNA, lipids, and proteins, due to reactive oxygen and nitrogen species (4). Numerous studies found that cognitive abilities can be safeguarded by consuming antioxidant-rich foods, one of which is tempeh (3, 5).

Tempeh, a fermented soybean product, is a traditional protein source known for its affordability, flavor, and digestibility in Indonesia. Produced through solid-state fermentation with *Rhizopus* spp., tempeh is made by soaking, dehulling, boiling, and incubating soybeans, forming a compact cake bound by mold mycelia during fermentation (6). Soybean fermentation enhances antioxidant properties by increasing vitamin B2, B12, and GABA levels. GABA, a neurotransmitter, regulates the central nervous system and improves cognitive function. Studies have shown that older people who consume tempeh have better memory due to its higher folate and B12 content, which may reduce the risk of dementia and Alzheimer’s disease (7).

Several studies have shown that soy tempeh significantly contributes to cognitive improvement. For instance, a study by Handajani et al. revealed that tempeh improves cognitive function. This mechanism is attributed to the presence of probiotics in tempeh, specifically *Lactobacillus fermentum* A2.8, which synthesizes gamma-aminobutyric acid (GABA), a neurotransmitter vital for cognitive function (8, 9).

Traditional single-ingredient tempeh can also be fortified with other legumes and seeds to improve its nutritional content (7). Sunflower seeds are known for their high antioxidant content, which promotes brain health and prevents neurodegenerative diseases. The fiber and antimicrobial properties of the seeds also contribute to overall health by reducing the risk of high blood pressure (10). Regular consumption of these seeds can aid in preventing memory-related conditions, such as dementia, while supporting cognitive health (11). Pumpkin seeds can protect brain cells due to their high antioxidant content. Not only are these seeds beneficial for memory, but they are also useful in alleviating depression and anxiety (12). Adzuki beans are highly popular in Asia due to their flavor and nutritional value. They are nutrient-dense and recognized for their high fiber and antioxidant content. Regular consumption of adzuki beans can offer protective benefits for diabetes prevention, kidney health, and cognitive function, potentially reducing the risk of degenerative brain diseases (13).

This study aims to evaluate the effects of mixed tempeh with sunflower seeds, pumpkin seeds, adzuki beans, and soybeans on the cognitive function of older people compared to soy tempeh. Most research projects have utilized soy tempeh, and the study involving mixed tempeh is the initial one to be carried out.

## 2 Materials and methods

### 2.1 Design study

This study used an experimental design.

### 2.2 Participants

The participants were gathered from the senior population, a target area for the Atma Jaya Aging Research Centre of Atma Jaya Catholic University of Indonesia. We took 360 elderly samples randomly from a total of 1,200. The inclusion criteria were participants with mild cognitive impairment (MCI) with Montreal Cognitive Assessment Indonesia (MoCA-InA) scores ranging from 18 to 25 or a score of MoCA-InA < 18 but with no impairment in Instrumental of Activities of Daily Living (IADL). Exclusion criteria included a significant diagnosis of diabetes or blood glucose levels greater than 200 mg/dl. During the consent and interview phases, the study participants were not living alone and were accompanied by family members who served as their caregivers.

**Figure 1:**
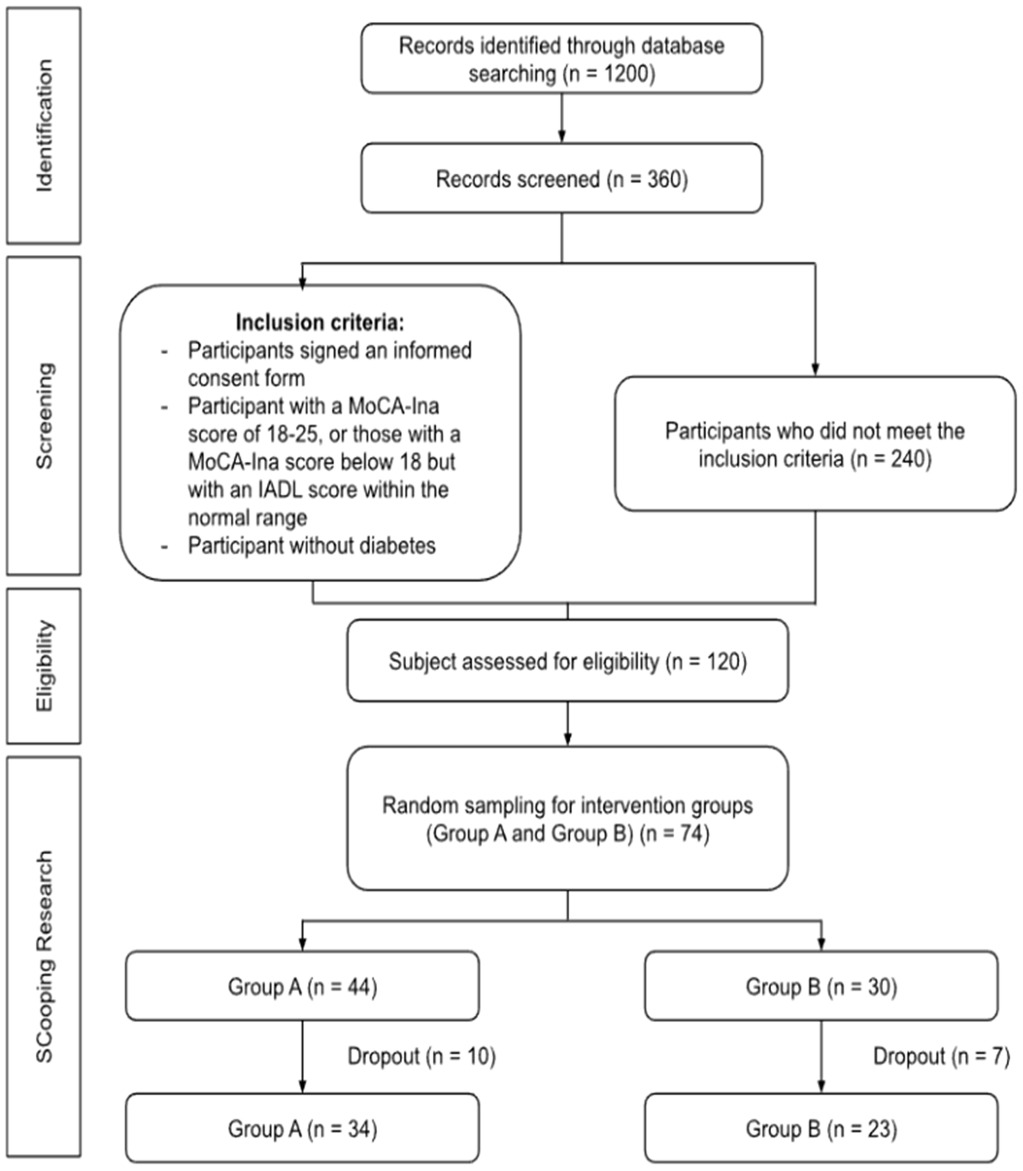
Consort flow chart of participants’ recruitment.

A total of 120 older people were eligible participants, and two groups of older people, group A and group B, were randomly selected without blinding for the intervention phase. Each group had 44 and 30 participants, for a total of 74 participants (alpha 5%; power 80%). The number of respondents who dropped out of this study was 17 older people (22.9%) for the following reasons: most were bored with daily tempeh consumption. In contrast, one respondent returned to their distant hometown, so the number of samples analyzed was 57 participants (Figure 1).

### 2.3 Tempeh intervention

#### 2.3.1 Tempeh group intervention

Participants were allocated into two intervention groups: mixed tempeh, comprising 44 subjects, and soy tempeh, comprising 30 subjects (Figure 1). The intervention entailed consuming 100 g of tempeh daily for 4 months.

Mixed tempeh consisted of a combination of sunflower seeds, pumpkin seeds, and adzuki beans, along with regular soybeans, in a ratio of 1:1:1:1. In contrast, soy tempeh was made solely from soybeans. Participants were given 100-g portions of tempeh and instructed to prepare it using methods such as steaming or stir-frying, while avoiding deep-frying to achieve a crisp texture. The portion size was based on previous research (14). To reduce potential biases from other dietary sources of soy protein or probiotics, participants agreed to refrain from consuming soy-based products or fermented foods, including tofu, soy milk, yogurt, and tape (fermented cassava), for the duration of the study. Local coordinators, who were informed about the study, managed the distribution of tempeh along with a monitoring card that served as both a calendar and a reminder for participants. This card was collected each month to assess daily adherence to the consumption guidelines.

#### 2.3.2 Materials of tempeh

The tempeh production process utilized four types of raw materials: soybeans (Tempeh K), pumpkin seeds (Tempeh TK), sunflower seeds (Tempeh TBM), and adzuki beans (Tempeh TA), each in equal ratios of 1:1:1:1. The production began with cleaning the raw materials to remove impurities, followed by washing them until the rinse water ran clear. Subsequently, the raw materials underwent the following steps: first soaking, first boiling, second soaking (after dehulling, specifically for soybeans), and second boiling, with different durations for each step (Table 1). After the raw materials were air-dried, they were inoculated with tempeh inoculum (2 g/kg of raw material) and incubated at 30°C for 42 h in perforated plastic packaging.

**Table 1:** The soaking and boiling duration of tempeh raw materials.

Types of raw materials	Soaking duration (hours)		Boiling duration (minutes)	
	First	Second	First	Second
Soybeans	2	24	20	15
Pumpkin seeds	2	24	20	15
Sunflower seeds	2	24	20	15
Adzuki beans	48	24	90	15

The bacteria isolated from mixed and soy tempeh were cultured on de Man, Rogosa, and Sharpe (MRS) Agar. After growth, the bacteria were harvested through centrifugation and washed three times with 0.85% NaCl to remove any residual media. For tempeh production, a bacterial concentration of 1 × 10^8^ colony-forming units (CFUs) was used. The tempeh bacteria were then analyzed using both conventional methods and metagenomic techniques.

### 2.4 Cognitive assessment

Cognitive assessments were conducted before and after the intervention, utilizing five cognitive domains; MoCA to evaluate the global cognitive domain alongside the Consortium to Establish a Registry for Alzheimer’s Disease (CERAD) toolkit, which includes the attention domain, memory domain (Word List Memory Immediate Recall (WLMIR), Word List Recall, and Word List Recognition tests), language domain (the Boston Naming Test (BNT) and verbal fluency), and visuospatial domain (15, 16). These assessments were selected to evaluate various cognitive regions within the brain. The IADL measure was taken to assess individual levels of dependency (17).

### 2.5 Statistics analysis

Statistical analysis was performed using IBM SPSS software version 27.0. We conducted univariate analysis to describe the demographic profile of the participants, utilizing percentages (%) for categorical variables (such as sex and education) and means with standard deviations (SD) for numerical data (such as age). All cognitive assessments were reported as numerical scores, and we compared the cognitive scores of subjects who received soy tempeh and mixed tempeh interventions both before and after the intervention. For each numerical dataset, we performed a normality test to determine the appropriate statistical analysis method (with a normality test *p*-value of ≥ 0.05). Variables that followed a normal distribution (specifically, WLMIR attempt 2) were analyzed using a paired t-test, while those that did not follow a normal distribution (including MoCA-InA, BNT, verbal fluency, visuoconstruction, WLMIR, Word List Recall, and Word List Recognition tests) were analyzed using the Wilcoxon test. A *p*-value of <0.05 was deemed statistically significant.

### 2.6 Ethical statement

The School of Medicine and Health Sciences, Atma Jaya Catholic University of Indonesia ethical committee approved the study, with referral number 08/07/KEP-FKIKUAJ/2024. All subjects were informed about the procedure and gave their written consent to participate.

## 3 Result

Fifty-seven respondents completed the study. The majority of respondents were female (68.4%), above 65 years of age (77.2%), and with an education level of 12 years or more (59.6%) (Table 2).

**Table 2:** Demography characteristics.

Variables	*N* total (%) = 57	Mixed tempeh	Soya tempeh
Demographic characteristics			
Age (Mean ± SD)	69.89 ± 5.04		
60–65 years old	13 (22.8)	7 (53.8)	6 (46.2)
>65 years old	44 (77.2)	27 (61.4)	17 (38.6)
Sex, (*n* %)			
Male	18 (31.6)	11 (61.1)	7 (38.9)
Female	39 (68.4)	23 (59.0)	16 (41.0)
Education, (*n* %)			
<12 years	23 (40.4)	12 (52.2)	11 (47.8)
≥12 years	34 (59.6)	22 (64.7)	12 (35.3)

SD: Standard deviation.

To assess the effects of the intervention, we compared the mean difference (MD) scores across all cognitive test scores. Statistically, the results showed that the mixed tempeh and soy tempeh groups significantly improved different cognitive domains. Mixed tempeh experienced a significant increase in mean difference (MD) before and after in three cognitive domains: global cognitive, memory (WLMIR1, WLMIR2, and Word list recall), and language (verbal fluency). On the other hand, soy tempeh experienced a significant increase in only two cognitive domains, which were memory (WLMIR1 and WLMIR2) and visuospatial (Table 3).

**Table 3:** Cognitive score differences before and after interventions in mixed and soya tempeh.

Variables	Mixed tempeh				Soya tempeh			
	Before	After	Mean difference	*p*-value	Before	After	Mean difference	*p*-value
Attention Domain	6.62 ± 1.30	6.18 ± 1.53	−0.441	0.190	5.43 ± 1.78	4.70 ± 1.58	−0.739	0.096
Global Cognitive Domain	22.71 ± 2.18	23.91 ± 2.68	1.206	0.006*	19.3 ± 3.65	20.7 ± 4.22	1.391	0.120
Memory Domain								
WLMIR (attempt 1)	3.12 ± 1.59	4.44 ± 1.91	1.324	0.002*	2.70 ± 2.07	3.74 ± 1.66	1.043	0.027*
WLMIR (attempt 2)	5.62 ± 1.62	6.26 ± 2.02	0.647	0.033**	4.39 ± 1.83	5.52 ± 1.59	1.130	0.025**
WLMIR (attempt 3)	7.18 ± 1.78	7.29 ± 1.51	0.118	0.709	5.78 ± 1.97	6.39 ± 1.80	0.609	0.249
Word List Recall	4.68 ± 2.37	5.56 ± 1.94	0.882	0.008*	3.87 ± 2.18	5.09 ± 2.22	1.217	0.059
Word List Recognition	17.56 ± 2.00	17.52 ± 2.21	0.058	0.729	16.70 ± 2.5	17.09 ± 2.63	0.391	0.599
Language Domain								
Boston Naming Test	13.38 ± 1.50	13.18 ± 1.60	−0.206	0.288	12.26 ± 2.26	12.65 ± 1.89	0.391	0.123
Verbal Fluency	15.97 ± 4.01	16.91 ± 3.94	0.941	0.045*	13.87 ± 4.28	15.35 ± 5.28	1.478	0.052
Visuospatial Domain	9.35 ± 1.61	9.44 ± 1.54	0.088	0.766	7.43 ± 2.73	8.74 ± 1.55	1.304	0.013*

*p = <0.05 using the Wilcoxon test. **p = <0.05 using the T-dependent test.

## 4 Discussion

In this study, we expect that consuming tempeh will improve cognitive function in older adults. Specifically, tempeh mixed with sunflower seeds, pumpkin seeds, and adzuki beans is anticipated to show better results than soy tempeh. Our research highlights the nutritional diversity offered by mixed tempeh.

When comparing the cognitive functions of the two groups, we found that mixed tempeh led to improvements across more cognitive domains than soy tempeh. Mixed tempeh consumption was associated with enhancements in three areas: global cognitive, memory, and language. In contrast, soy tempeh showed improvements in only two areas: memory and visuospatial. Although both types of tempeh improved memory function, mixed tempeh demonstrated a greater enhancement in this area. Notably, mixed tempeh increased word list recall, indicating better memory retention than soy tempeh. Additionally, mixed tempeh showed improvements in language functions. A study conducted by Handajani et al. (18) found that subjects with impairments in language, global cognition, and word list memory recall impairment were at a significantly higher risk—over 4.5, 2.2, and 2.8 times, respectively—for developing frailty (18). Memory and language functions are critical cognitive domains often affected in older individuals with degenerative diseases (19).

Several studies have identified the benefits of various forms of soy tempeh, including traditional tempeh, supplements, and probiotic beverages, all of which have demonstrated positive effects on cognitive improvement (8, 9, 20).

The potential cognitive benefits of mixed tempeh in this study may stem from its diverse nutritional content, with each ingredient providing complementary nutrients. Sunflower seeds are particularly rich in essential nutrients, including protein, healthy fats, fiber, vitamins (especially vitamin E), selenium, copper, zinc, folate, and iron (21). Moreover, sunflower seeds contain beneficial compounds such as phenolics, flavonoids, and polyunsaturated fatty acids (22). They also provide several essential amino acids, including aspartic acid, glutamic acid, serine, histidine, glycine, threonine, arginine, alanine, tyrosine, cysteine, valine, methionine, phenylalanine, isoleucine, leucine, lysine, and proline (23). Guo et al. reported that sunflower seeds contain various phytochemical compounds that exhibit multifunctional biological activities (24).

Adzuki beans are another valuable ingredient, offering high-quality protein (25). They are also rich in minerals, such as calcium, magnesium, and zinc, and vitamins, such as tocopherol and folate (25, 26). Additionally, adzuki beans contain bioactive compounds, including saponins (27).

Pumpkin seeds contribute healthy polyunsaturated fatty acids, particularly linoleic acid (omega-6) and alpha-linolenic acid (omega-3) (28). Omega-3 fatty acids have been reported to stimulate neurogenesis in adults (29).

Regardless of the diversity of microorganisms formed due to the fermentation process in mixed tempeh, sunflower seeds, pumpkin seeds, and adzuki beans are widely recognized for their health benefits. Sunflower seeds, pumpkin seeds, and adzuki beans are particularly noteworthy, with sunflower seeds being especially rich in proteins, unsaturated fats, vitamins, and essential minerals. Sunflower seeds are an excellent source of antioxidants, including vitamin E, which helps prevent oxidative damage and provides anti-inflammatory, cardiovascular protective, and anticancer effects. The anti-inflammatory properties of sunflower seeds are associated with specific bioactive peptides (DVAMPVPK and PADVTPEEKPEV) that activate the Keap1/Nrf2 signaling pathway. This mechanism helps regulate the body’s response to oxidative stress (30). Additionally, sunflower seeds have significant neuroprotective effects due to their high content of tocopherols and unsaturated fatty acids. These components may aid in preventing ischemic brain injury, stroke, and cognitive decline (11). For example, a study conducted on mice found that pretreatment with sunflower oil significantly reduced brain lipid peroxidation, infarct volume, and post-ischemic edema, with an effective dose determined to be 3 ml/kg (31).

Dietary inclusion of sunflower seeds has been linked to improved memory and cognitive function, potentially delaying the onset of Alzheimer’s disease. This benefit is attributed to increased levels of brain phosphatidylcholine, a phospholipid essential for myelination, synaptic integrity, and neurotransmitter synthesis (11). Additionally, a diet enriched with sunflower oil has been shown to significantly elevate levels of docosahexaenoic acid (DHA) and arachidonic acid (AA) (*p* < 0.05), which are two essential fatty acids that are highly concentrated in the brain (32). In a human study, supplementation with sunflower seeds (50 mg/kg) alongside black mulberry and pumpkin seeds over 2 months improved memory in young adults. This cognitive enhancement was associated with increased neuroplasticity, as indicated by elevated levels of brain-derived neurotrophic factor (BDNF) and reduced levels of the stress hormone glucocorticoid receptor-α. These findings highlight the potential of sunflower seeds as a nutritional intervention to support and enhance brain health (33).

Pumpkin seeds offer several cognitive benefits due to their high content of tocopherols, particularly γ- and β-tocopherol. These compounds naturally neutralize free radicals and may help prevent cardiovascular diseases (33). Additionally, consuming pumpkin seeds can promote greater probiotic diversity in the gut, reduce pathogenic bacterial populations, and enhance intestinal permeability. These factors are all essential for supporting neurological function and maintaining a healthy gut-brain axis (34).

Recent research has also identified lycopene, a carotenoid compound found in pumpkins, which plays a role in the expression of Electron Transport Chain Complex I within the blood–brain barrier of neuronal cells. This suggests that lycopene may help regenerate mitochondrial function, potentially improving brain health, especially in individuals with Autism Spectrum Disorder (34, 35).

Furthermore, a randomized controlled trial examining the effects of brain-boosting foods—specifically substituting sugar with pumpkin seeds—demonstrated its effectiveness when implemented within the framework of the National Health Service (36).

Adzuki beans are rich in dietary fiber and essential minerals, including iron, calcium, phosphorus, manganese, and sulfur, particularly concentrated in their seed coats. They also contain a variety of phytochemicals with antioxidant, anti-inflammatory, and antibacterial properties, contributing to their extensive health benefits (37). Among these compounds, saponins and flavonoids may significantly promote cognitive enhancement and support weight loss. Notably, vitexin and its isomer, isovitexin, are particularly linked to cognitive improvement, as they have been shown to reduce memory impairment induced by scopolamine in animal models (13).

Research indicates that adzuki beans can help mitigate obesity-related cognitive decline. For instance, adzuki bean extract significantly improved spatial learning and recognition abilities in mice exposed to a high-fat diet. These mice showed a strong preference for exploring novel objects and routes, as demonstrated by T-maze and novel object recognition tests (38). Moreover, incorporating adzuki bean extract into the diet markedly increased life expectancy, with Drosophila models exhibiting an extension of over 10 days. The extract also appears to reduce the aggregation and deposition of amyloid-beta (Aβ42) in the brain, which is closely associated with the cognitive symptoms of Alzheimer’s disease. In Aβ42-overexpressing flies, adzuki bean extract alleviated memory dysfunction by inhibiting Aβ42 aggregation and reducing oxidative stress induced by Aβ42 proteins (39). Overall, our study has shown the diverse benefits of mixed tempeh and the role of adzuki beans in promoting health.

In contrast to mixed tempeh, numerous studies have identified soy tempeh as beneficial for cognitive improvement. A study by Handajani et al. (9) conducted a 6-month intervention where older participants with mild cognitive impairment (MCI) consumed 100 g of tempeh daily. The study found significant improvements in cognitive functions. Additionally, Handajani et al. (8) reported enhancements in memory, language, and visuospatial functions among older individuals who were given probiotics derived from *L. fermentum* isolated from tempeh at concentrations of 108 and 107 CFU/ml. Notably, improvements in the learning process were more pronounced at 108 CFU/ml. Another study by Hwang et al. indicated that supplementation with soybean fermentation products enhanced attention, working memory, and verbal memory (20).

A systematic review of the neuroprotective effects of fermented dietary intake in older adults found that daily consumption of soy products is linked to significant improvements in cognitive function, suggesting their potential benefits for brain health (40). Recent scientific studies indicate that the bioactive compounds present in tempeh—such as antioxidants and anti-inflammatory agents—positively impact cognitive wellbeing (8).

Animal studies have also highlighted the neuroprotective effects of soybean and tempeh. Research by Hamad et al. revealed that extracts from both soybean and tempeh improved memory, with tempeh displaying greater effects (*p* < 0.05) (41). Tempeh significantly increased acetylcholine (ACh) levels and reduced inflammation more effectively than soybean. Furthermore, another study noted increased activity of catalase and superoxide dismutase (SOD) in the cortex, hippocampus, and striatum of SAMP8 mice after tempeh consumption, demonstrating its cognitive and oxidative benefits (42).

Several potential mechanisms may explain improvements in cognitive functions. One notable mechanism involves increased brain-derived neurotrophic factor (BDNF) levels. Hwang et al. observed that the cognitive enhancements in their subjects following supplementation with soybean fermentation products were positively correlated with elevated serum BDNF levels. Additionally, butyrate, a short-chain fatty acid produced by butyric acid-producing bacteria found in probiotics, was identified as a compound that boosts BDNF secretion (20).

The fermentation of soybeans has led to the development of various fermented soy products that exhibit antioxidant properties. These products contain increased levels of vitamin B2, vitamin B12, and gamma-aminobutyric acid (GABA). Numerous studies have underscored the effectiveness of these vitamins, with GABA specifically linked to the regulation of the central nervous system and improvements in cognitive function (43, 44).

Moreover, fermented tempeh extracts—especially those co-fermented with Rhizopus and Lactobacillus—have been shown to contain higher amounts of GABA and anthocyanins. These compounds have demonstrated neuroprotective effects on BV-2 microglial cells, a type of brain cell stimulated by lipopolysaccharides (LPS), which trigger inflammation and are associated with various health issues. The neuroprotective effects were achieved by reducing the activity of nitric oxide synthase and reactive oxygen species (ROS) while also lowering the expression of the phospho-cyclic AMP response element-binding protein gene and increasing the expression of genes related to neurotrophic factors (45).

The study has several notable limitations. First, the probiotics used in tempeh production and the protein content of the two groups were not standardized. This lack of standardization makes it challenging to determine whether the observed improvements were due to differences in antioxidants, probiotics, or protein content. Additionally, the intervention was conducted without blinding, allowing participants to visually distinguish between the mixed tempeh, which appeared darker, and the soybean tempeh. The limited number of research participants also hindered the analysis of tempeh’s benefits related to demographic variables. Furthermore, the study had a high dropout rate, which resulted in a smaller sample size. Future research should evaluate biomarkers of antioxidant activity, such as total antioxidant capacity assays, to gain a more accurate understanding of the role of antioxidants in cognitive function.

In conclusion, the study highlights the cognitive benefits of tempeh, particularly when mixed with other nutrient-rich ingredients. The combination of sunflower seeds, pumpkin seeds, and adzuki beans in mixed tempeh provides superior neuroprotective effects compared to traditional soybean-based tempeh. This research supports the idea of promoting mixed tempeh as a healthy food alternative, especially for older adults, by offering enhanced nutritional value and cognitive health benefits.

## Data Availability

The raw data supporting the conclusions of this article will be made available by the authors, without undue reservation.
